# A Novel Homozygous *PKP2* Variant in Severe Neonatal Non-compaction and Concomitant Ventricular Septal Defect: A Case Report

**DOI:** 10.3389/fped.2021.801491

**Published:** 2022-01-04

**Authors:** Poomiporn Katanyuwong, Arthaporn Khongkraparn, Duangrurdee Wattanasirichaigoon

**Affiliations:** ^1^Division of Cardiology, Department of Pediatrics, Faculty of Medicine Ramathibodi Hospital, Mahidol University, Bangkok, Thailand; ^2^Division of Medical Genetics, Department of Pediatrics, Faculty of Medicine Ramathibodi Hospital, Mahidol University, Bangkok, Thailand

**Keywords:** *PKP2*, ventricular non-compaction, arrhythmogenic right ventricular dysplasia/cardiomyopathy (ARVD/C), hypoplastic left heart syndrome, case report

## Abstract

Left ventricular non-compaction (LVNC) is a rare and genetically heterogeneous cardiomyopathy. The disorder vastly affects infants and young children. Severe neonatal LVNC is relatively rare. The prevalence of genetic defects underlying pediatric and adult-onset LVNC is about 17–40%. Mutations of *MYH7* and *MYBPC3* sarcomeric genes are found in the vast majority of the positive pediatric cases. *PKP2* encodes plakophilin-2, a non-sarcomeric desmosomal protein, which has multiple roles in cardiac myocytes including cell–cell adhesion, tightening gap junction, and transcriptional factor. Most of the reported *PKP2* mutations are heterozygous missense and truncating variants, and they are associated with an adult-onset autosomal dominant disorder, namely arrhythmogenic right ventricular dysplasia/cardiomyopathy (ARVD/C). Homozygous *PKP2* mutations have been rarely described. Herein, we present a rare case of an infant with neonatal onset of congestive heart failure owing to severe LVNC and multiple muscular VSD. Medical treatments failed to control the heart failure and the patient died at 11 months of age. Whole-exome sequencing identified a novel homozygous *PKP2* variant, c.1511-1G>C, in the patient. An mRNA analysis revealed aberrant transcript lacking exon 7, which was predicted to cause a frameshift and truncated peptide (p.Gly460GlufsTer2). The heterozygous parents had normal cardiac structures and functions as demonstrated by electrocardiogram and echocardiography. Pathogenic variants of sarcomeric genes analyzed were not found in the patient. We conducted a literature review and identified eight families with biallelic *PKP2* mutations. We observed that three families (our included) with null variants were linked to lethal phenotypes, while homozygous missense mutations resulted in less severe manifestations: adolescent-onset ARVD/C and childhood-onset DCM. Our data support a previous notion that severe neonatal LVNC might represent a unique entity and had distinct genetic spectrum. In conclusion, the present study has extended the phenotypes and genotypes of *PKP2*-related disorders and lethal LVNC.

## Introduction

Ventricular non-compaction is a rare cardiomyopathy with genetic heterogeneity. It is characterized by prominent trabeculation and deep intertrabecular recesses. The disorder constantly affects the left ventricle, the so-called LVNC, with involvement of the right ventricle in 22–43% of cases (1, 2). LVNC is diagnosed based on the established criteria of echocardiography and/or magnetic resonance imaging (MRI) (2–4). It is believed that pediatric LVNC results from developmental arrest, which leads to persistence of a trabecular network of spongy ventricular muscle, during mid-to-late embryonic stages (2). The disorder mostly affects infants and young children, with progressive clinical course. LVNC with perinatal manifestations is relatively rare. Contrarily, adult-onset LVNC is potentially reversible, non-embryogenic, and with a less progressive clinical course (2).

Genetic defects underlying pediatric and adult-onset LVNC have indicated a 17–40% positive rate (5). Most cases of LVNC are caused by mutations of sarcomeric genes, including autosomal dominant variants of *MYH7* and *TTN* and autosomal recessive alleles of *MYBPC3* (2, 5). However, only mutations of *MYH7* and *MYBPC3* accounts for the vast majority of pediatric cases, while variants of X-linked mitochondrial disorder-related genes and chromosomal abnormalities are responsible for the remainder of cases (2, 5, 6).

*PKP2* encodes plakophilin-2, a non-sarcomeric desmosomal protein, which plays major roles in cell–cell adhesion, tightening gap junction, intracellular signaling, electrophysiologic regulation, protein trafficking, and transcriptional factor activity (http://www.uniprot.org). Over 350 missense and truncating *PKP2* variants have been described to be a major (40–60%) cause of arrhythmogenic right ventricular dysplasia/cardiomyopathy (ARVD/C), which is an adult-onset autosomal dominant disorder characterized by progressive fibrofatty infiltration of myocardium and pre-disposing to syncope, ventricular arrhythmias, and sudden cardiac death in young adults (7).

Homozygous *PKP2* mutations have been rarely described. In this study, we present a rare occurrence of concomitant severe neonatal LVNC and muscular VSD with neonatal onset of cardiac failure, including a novel homozygous *PKP2* variant and its molecular consequence. Previously reported biallelic mutations of *PKP2* are also briefly reviewed.

## Case Report

### Clinical Data

The patient was a 40-week gestational age female infant, with birth weight of 3,240 g, following an uneventful pregnancy. She had tachycardia, tachypnea, and heart murmur after birth. Chest roentgenogram showed marked cardiomegaly. Echocardiogram revealed large multiple muscular VSD, biventricular hypertrophy with ventricular non-compaction morphology. The patient had an impaired left ventricular systolic function (LVEF = 40%). Medical treatments at the birth hospital (a tertiary hospital in the region) were initiated to control the congestive heart failure; these included dobutamine, digoxin, and furosemide. After her heart failure was under control, she was discharged home on day of life 47. It was reported that the patient had regular follow-up at the hospital, without further hospitalization. On the last visit at the age of 5 months, the patient appeared to have failure to thrive and worsening heart failure, prompting the referral to our institution for further investigation and management.

**Figure 1:**
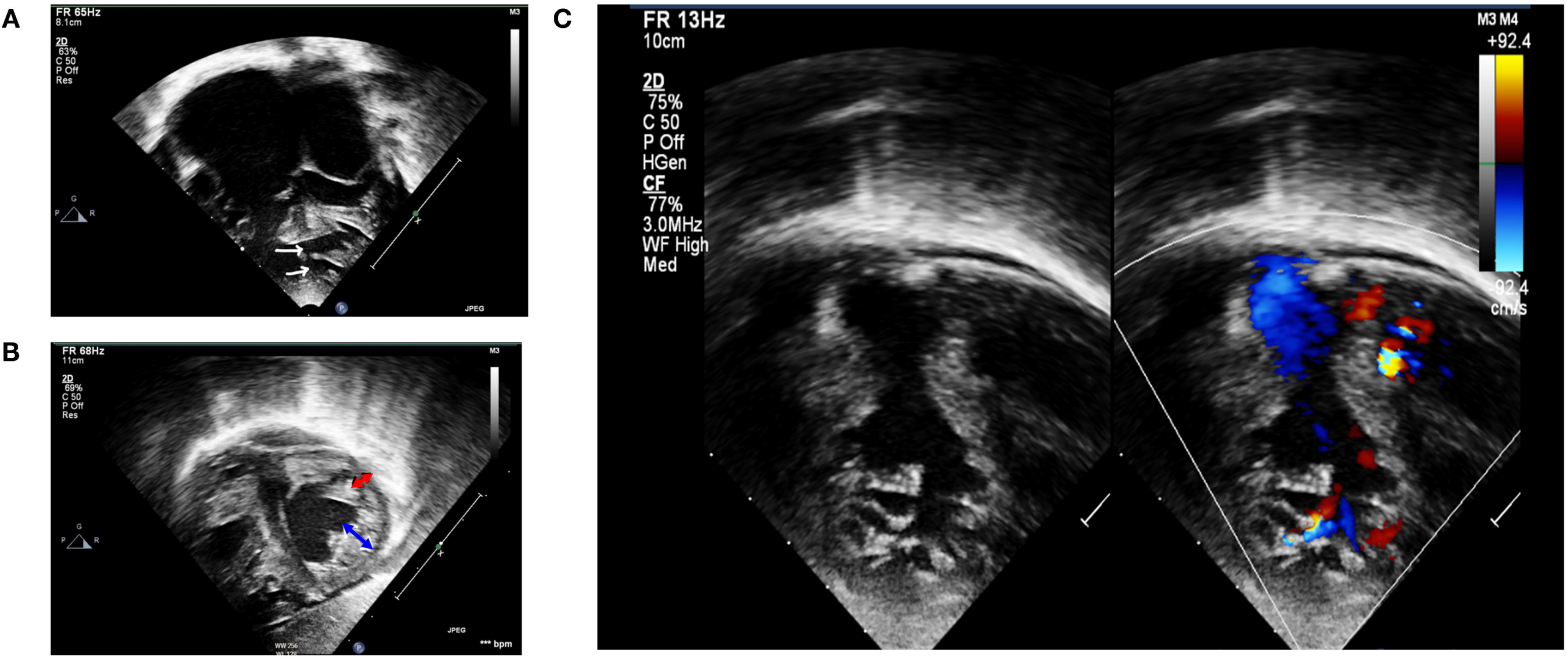
Transthoracic echocardiography. **(A)** Apical four-chamber view showing biventricular hypertrophied heart with multiple mid and apical-muscular VSD (white arrows); markedly dilated atria, especially the right atrium. **(B)** Subcostal short-axis view in end-systole revealing non-compaction cardiomyopathy involving both ventricles. Non-compaction area is depicted by blue arrow, whereas compaction area is depicted by red arrow. **(C)** Left panel: 2D echocardiography demonstrating RV non-compaction with multiple and prominent trabeculation with deep recesses; right panel: a color Doppler echocardiography demonstrated direct communication between RV cavity into deep intertrabecular recesses.

Physical examination (at 5 months) at our institution revealed a cachexic infant with a body weight of 4,200 g (<3rd centile), height of 60 cm (10th centile), RR of 60/min, HR of 120/min, and SpO_2_ at 99%. There was active precordium, loud P2, holosystolic murmur grade III/VI at the left lower sternal border, and hepatomegaly. A 12-lead EKG revealed a sinus rhythm, right axis deviation, biatrial enlargement, and biventricular hypertrophy. We did not detect any arrhythmia or conduction abnormality, including wide QRS complex and Brugada pattern from telemetry, while the patient was in the ICU. Echocardiogram confirmed non-compaction cardiomyopathy (NC:C ratio > 2) involving both RV and LV, severe LV systolic dysfunction with LVEF of 26–28%, impaired RV systolic function with TAPSE of 5 mm, bilateral enlargement, and multiple muscular VSD located at the mid and apical regions of the ventricular septum (Figure 1). Medical treatment was optimized by adding spironolactone for the control of congestive heart failure and a low dose of aspirin for prevention of thromboembolic complications. Nutritional managements, including high caloric formula, were also added. The congestive heart failure of the patient was difficult to control, owing to the severely impaired LV systolic function and the multiple VSD.

On the last follow-up at our institution, at 9 months of age, the heart failure of the patient appeared to be worsening with persistent tachypnea and poor weight gain, requiring hospitalization to control the cardiac failure and for supportive care. A repeated echocardiogram revealed multiple muscular VSD at the mid and apical part of the ventricular septum. There was pulmonary hypertension secondary to VSD and ventricular non-compaction. Pulmonary systolic pressure was measured at 80 mmHg using tricuspid regurgitation Doppler. The LV systolic function was improved compared with the last time, with an LVEF of 50%. Due to severe pulmonary hypertension, we planned to perform cardiac catheterization to assess pulmonary vascular resistance and the possibility of successful surgical VSD closure, which could prevent the development of Eisenmenger physiology. As for the biventricular non-compaction, cardiac MRI was planned to evaluate RV function, while the medical treatments were continued. Our institution established a pediatric heart transplantation program in 2019. Since then, we have performed two pediatric heart transplants with great success; however, the procedure has been limited to a child weighing >30 kg due to our limited capability. There was no other institution in the country that offered pediatric heart transplant for a child with that young age. Therefore, conservative treatment and palliative care were discussed with the family, and the patient was discharged home at 10 months of age, with full medications.

At the age of 11 months, the patient presented to the local hospital with fever and tachypnea, and was diagnosed with pneumonia. The condition aggravated her cardiac symptoms and necessitated intubation and ventilatory support, eventually resulting in death shortly after. Cardiac MRI and autopsy were not done. After a month, we were notified by the parents about the unfortunate event of the child. Subsequently, the result of genetic testing became available, and genetic counseling was provided to the family.

The mother and the father were aged 22 and 23 years, respectively. The couple originated from the same district, but they had no known consanguinity. Echocardiograms and electrocardiograms of the parents revealed normal cardiac structures and functions.

### Genetic Data

Peripheral blood samples from the patient and the parents were obtained for genetic analysis, after written informed consent was given. DNA was extracted from the blood samples using a Gentra® Puregene® kit (QIAGEN®, Hilden, Germany).

Briefly, whole-exome sequencing of the patient's specimen was performed on Illumina HiSeq2500 by Macrogen® (Seoul, Republic of Korea) and analyzed using previously described methods (8) and human phenotype ontology terms, cardiomyopathy (HP:0001638), which included 433 genes of sarcomeric and non-sarcomeric proteins. PCR-Sanger sequencing was performed to validate the identified variant.

**Figure 2:**
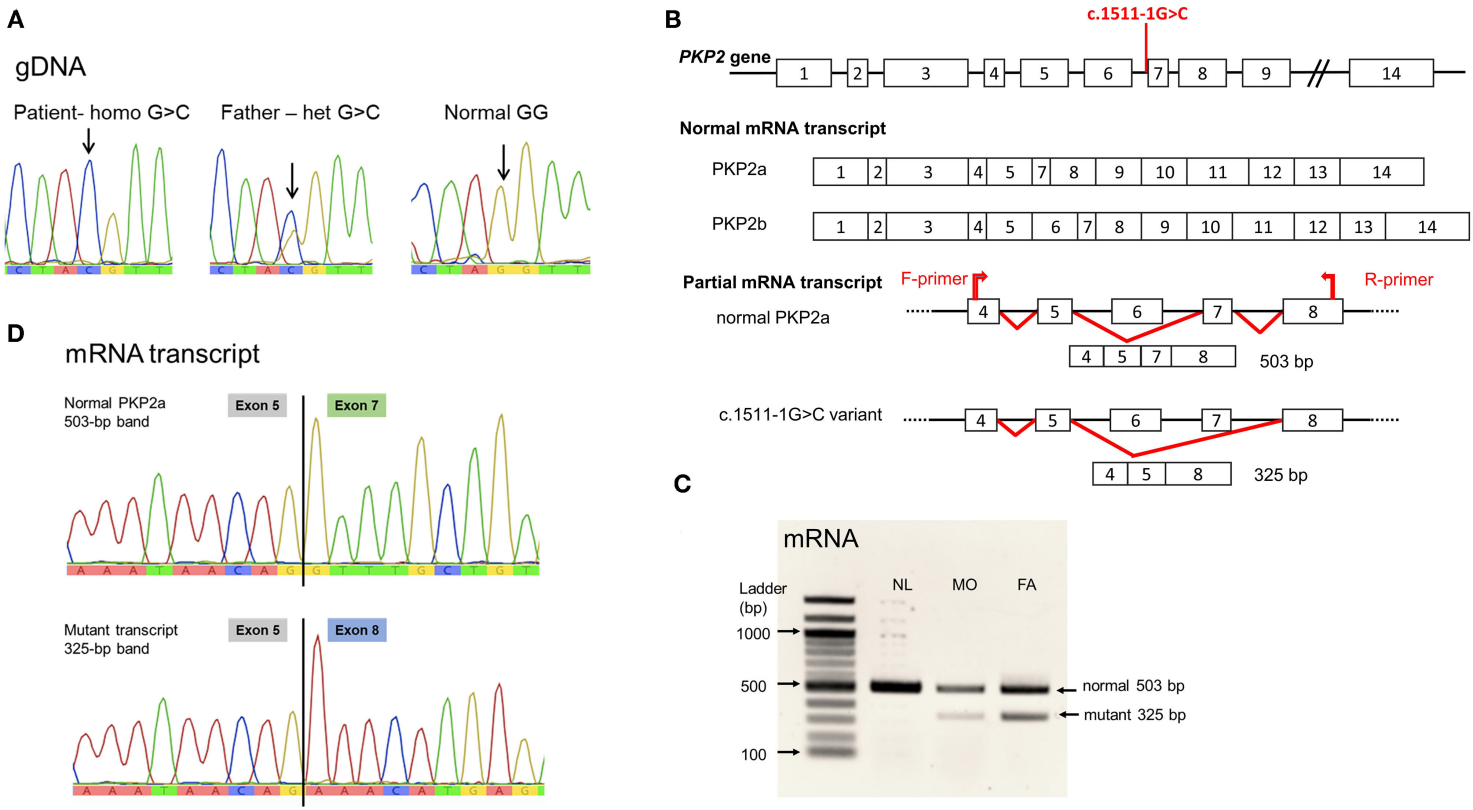
*PKP2* variant, c.1511-1G>C. **(A)** Genomic DNA sequence. Noted nucleotide G/G in normal control, G/C in heterozygous father, and C/C in the patient. **(B)** Diagram showing the location of the variant at intron 6 acceptor site; normal PKP2 mRNA transcript isoform 2a lacking exon 6; and the mutant variant eliminating exon 7. **(C)** Agarose gel showing the mRNA (cDNA) detected. Noted both normal size, 503 bp, and the mutant, 325-bp, mRNA transcripts in the heterozygous father (FA) and mother (MO); and only normal-sized mRNA in the normal control (NL). **(D)** mRNA sequences. Normal PKP2a mRNA transcript with sequence of exon 5 joined by exon 7; mutant transcript lacking exon 7, thus, exon 5 connected to exon 8 sequence. Sequence of exon 6 was not present in PKP2a isoform.

The WES analysis revealed a novel homozygous nucleotide substitution at the splice acceptor site of intron 6 of the *PKP2*, c.1511-1G>C, or IVS6-1G>C, in the patient. Both parents were heterozygous for the allele (Figure 2A). This variant was classified as pathogenic (PV1, PM2, and PP3) according the 2015 guidelines of the American College of Medical Genetics and Genomics and the Association of Molecular Pathology (ACMG/AMP) (9). The variant was not present in population databases, including gnomAD (MAF = 0; https://gnomad.broadinstitute.org/) and the Thai Reference Exome Database (MAF 0/2000; T-REx, https://trex.nbt.or.th/) and disease databases including ClinVar and the Human Gene Mutation Database (HGMD). The variant was submitted to ClinVar and received a reference number, SCV001451920.

The splicing defect of c.1511-1G>C variant was evaluated using *in silico* analysis software, NNSPLICE 0.9 (https://www.fruitfly.org/seq_tools/splice.htm), which indicated the loss of splicing ability, with a score reduced from 0.96 to 0, resulting in exon 7 skipping. Parental blood samples were used for mRNA analyses because of no available specimens of the patient, following established protocols (8). PCR of the mRNA transcript of coding exons 4–8 yielded normal-sized and aberrant transcripts in the specimens of the parents, with notably less abundance of the mutant mRNA (Figures 2B,C). Sanger sequencing demonstrated the lack of exon 7 in the mutant transcript (Figure 2D).

The detailed methods of WES analysis, primer sequences, and mRNA study were provided as Supplementary Data. Reference sequences were NT_009714, NM_001005242.3, and NM_001005242 (for PKP2a isoform).

Additionally, heterozygous variants of two distinct sarcomeric genes were identified in the patient's specimen, including *MYH7*:c.732C>T and *TNNT2*:c.53-11_53-7delCTTCT. Both variants were classified as benign in ClinVar and according to the ACMG/AMP guidelines as it had high MAF in the genomAD: c.732C>T with MAF 0.175 and c.53-11_53-7delCTTCT with MAF 0.602.

We also reviewed previously reported biallelic mutations of *PKP2*, with just eight families described in the literature, as summarized in Table 1 (10–16).

**Table 1:** Clinical characteristics of families reported with biallelic *PKP2* variants.

**Family**	**Pt**.	***PKP2* variant**	**Age at presentation**	**Presenting manifestations**	**Outcome and specific treatment**	**Heterozygous parents' phenotypes**	**References**
1	1.1^a^	hom c.1211dup (p.Val406fsTer4)	38 weeks GA	Fetal hydrops, HLHS, severe RVH, NC, multiple VSD	IUFD at 38 weeks GA	None at ages 28 and 30 years, but signs of ARVC at ages 46 and 48 years	(10)
	1.2^a^		Prenatal	Fetal hydrops, HLHS, reduced contractility of both ventricles, RVH with NC	Dead, DOL19		
2	2.1^b^	hom whole *PKP2* gene deletion (1.2 Mb)	32 weeks GA	Fetal hydrops, cardiac failure, diffused biventricular NC	Dead, DOL12	None at ages 35 and 41 years (by cardiac MRI)	(11)
	2.2^b^		29 weeks GA	Cardiac failure, ventricular NC	TOP		
3	3	hom c.2484C>T^c^	Late teens	ARVD	ICD implanted at the age of 44	None at ages 76, 73, 17, 15, and 11 years (5 persons)	(12)
4	4.1	c.259G>C/c.587InsG (p.Val87Leu/ p.Ser197PhefsTer19)	33 years	ARVD		None	(13)
	4.2		34 years				
	4.3		62 years				
5	5	hom c.2489+1G>A (IVS12+1G>A)	36 years	ARVD	ICD implanted	None	(13)
6	6	hom c.2577+1G>T (IVS13+1G>T)	32 years	Syncope, ARVD	ICD implanted	None	(14)
7	7	hom c.1592T>G (p.Ile531Ser)	Young adult	ARVD	NA	None	(15)
8	8.1^d^	hom c.2035C>T (p.His679Tyr)	Early childhood onset	DCM: advanced disease	Early heart transplantation at age 14, 13, and 17 years	None	(16)
	8.2^d^						
	8.3^d^						
9	9	hom c.1511-1C>C (p.Gly460GlufsTer2)	At birth	Cardiac failure, biventricular NC, BVH	Dead at 11 months	None at age 22 and 24 years	Present family

a, b *Siblings*.

c *mRNA analysis revealing mixed wild-typed transcript and aberrant transcript due to splicing errors*.

d *Siblings and having family history of sudden cardiac death in three other untested young sibs, but also having an unaffected sib with homozygous variants*.

*RVD, arrhythmogenic right ventricular dysplasia; DCM, cardiomyopathy; DOL, day of life; GA, gestational age; het, heterozygous; HLHS, hypoplastic left heat syndrome; hom, homozygous; ICD, implantable cardioverter-defibrillator; IUFD, intrauterine fetal death; NA, not available; NC, non-compaction; RVH, right ventricular septal defect; TOP, termination of pregnancy; VSD, ventricular septal defect*.

## Discussion

We identified a novel pathogenic homozygous variant of *PKP2* as the probable cause of severe neonatal LVNC and concomitant muscular VSD. The severe congestive heart failure at birth was likely due to the large and multiple muscular VSD resulting in systemic pulmonary hypertension and the non-compaction cardiomyopathy affecting systolic and diastolic functions of both ventricles. The markedly dilated atria, especially the right atrium, strongly suggests severe tricuspid regurgitation, high end-diastolic pressures, restrictive physiology, and squared with the *PKP2* variant.

As we did not perform an analysis of genomic structural variant using whole-exome sequencing data or cytogenomic microarray, structural variant (if there is any) cannot be completely ruled out as the cause of VSD in the present case. However, we believe that the chance would be quite low, given the absence of dysmorphic features (as examined by a clinical geneticist, DW) and extracardiac abnormalities, which are frequent associated findings in such case. Moreover, multiple or Swiss cheese muscular VSD is not a common finding in the vast majority of VSD (17), while this specific type of VSD tends to be found associated with LVNC (1, 2, 18).

Although there was no history of parental consanguinity, the inheritance of the same variant of the c.1511-1G>C allele could be a founder mutation in the region.

*PKP2* comprises 14 exons and yields two isoforms of mRNA transcripts, PKP2a and PKP2b. The PKP2a is a major isoform expressed in the heart and also in peripheral blood. This isoform does not contain the exon 6 sequence. It has 2,514 bp of open reading frame (ORF), which is translated into a 837-aa peptide (http://genatlas.medecine.univ-paris5.fr/) (19). The other isoform, PKP2b, is rarely detectable. In the present study, only the PKP2a isoform was detected and referred to as PKP2.

The splice site mutation *PKP2*:c.1511-1G>C was predicted to cause skipping of exon 7, and the mutant transcript was confirmed by mRNA study. The frameshift mutation led to a new reading frame, resulting in a substitution of glycine by glutamic acid at codon 460, followed by a short frameshift before pre-mature termination two residues after (designated p.Gly460GlufsTer2).

The marked reduction of aberrant transcript in the heterozygous parents with c.1511-1G>C allele likely indicates the activation of mRNA decay, leading to null protein and resulting in no clinical phenotype in heterozygous individuals. On the other hand, the homozygous c.1511-1G>C mutation would lead to nearly a total absence of the PKP2 transcript and protein, causing the lethal phenotypes as seen in the present patient. Our findings are in agreement with the study in knock-out mice that homozygous null allele resulted in lethal cardiomyopathy, including reduced trabeculation of the ventricles, molecularly reduced architectural stability of the intercalated disks, disarrayed cytoskeleton, and ruptures of cardiac walls (20).

Based on our review of the literature, there were only nine *PKP2* variants present in biallelic mutations, seven of which were null variants, including small insertion/deletions (indels), whole-gene deletion, and splicing defects (Table 1) (10–16). The null alleles were associated with severe lethal phenotypes, namely, hypoplastic left heart syndrome (HLHS) and ventricular non-compaction with perinatal onset of cardiac failure (families 1, 2, and 9). Instead, homozygous missense *PKP2* variants led to relatively less severe manifestations: adolescent-onset ARVD/C and childhood-onset DCM (families 7 and 8). Those with splicing mutations showed variable phenotypes of earlier-onset ARVD/C in adults (families 3, 4, 5, and 6) and lethal presentation (family 9). Intrafamilial variabilities were not apparent in the families with bialleic *PKP2* mutations.

A recent study of a large cohort of LVNC in fetal population showed a positive genetic test at 47% (9/20) and that mutations of non-sarcomeric genes accounted for the vast majority, including *de novo* dominant mutations of *KCNH2* and *PRKAG2* genes and maternally inherited X-linked gene mutations of *NONO*, with the exception of one case harboring a *de novo* mutation of a sarcomeric gene, *TPM1* (21). The authors proposed that LVNC in fetal population with intrauterine onset heart failure might represent a unique entity and had distinct genetic spectrum (3). Our data support their findings that the sarcomeric genes including *MYH7* and *MYBPC3* may not be the major responsible genes underlying LVNC in this exceptional population.

Ventricular septal defect (VSD) is a common congenital heart disease. In most (75–80%) cases, the defects represent a perimembranous type, whereas the remainder are muscular VSDs, located at the apical to mid interventricular trabecular septum (17). The prevalence of CHD in LVNC was 20–50% depending on recruitment criteria, with VSD being the most common associated CHD (27–35%), followed by Ebstein's anomaly, atrial septal defect, and transposition of great arteries (1, 2, 6, 18, 22). When focusing on severe perinatal-onset LVNC, the higher prevalence of VSD (37–62%) was observed (21–23) and that apical muscular subtype appeared to be an unusually frequent finding compared with the general low prevalence, 10–20% (17).

The strength of this study was the confirmation of altered mRNA transcription and the expanding genotype related to neonatal/perinatal LVNC. The study contains some limitations as follows: the absence of the cardiac MRI and/or autopsy of the patient to illustrate an in-depth pathological detail; the absence of parental cardiac MRI, which may be superior over echocardiogram in demonstrating fibrofatty change representing ARVD/C or subclinical cardiomyopathy; and the absence of the mRNA data of the patient, which would be a more authentic *in vivo* evidence of the altered mRNA transcription in the homozygous individual.

## Conclusion

A novel homozygous PKP2 variant was identified as the probable cause of severe neonatal LVNC and concomitant VSD. Our data support the previous notion that severe neonatal LVNC might represent a unique entity and had distinct genetic spectrum. In conclusion, this study has expanded the phenotypes and genotypes of *PKP2* disease and lethal LVNC.

## Data Availability Statement

The original contributions presented in the study are included in the article/Supplementary Materials, further inquiries can be directed to the corresponding author.

## Ethics Statement

The studies involving human participants were reviewed and approved by the Ramathibodi Hospital Human Research Ethics Committee (Protocol ID 2006: MURA2020/1772 and ID 2379: MURA2021/42). Written informed consent to participate in this study was provided by the participants' legal guardian/next of kin.

## Author Contributions

PK provided clinical care, analyzed clinical data, and wrote the manuscript draft. AK performed molecular and bioinformatics analysis and wrote part of the manuscript. DW obtained funding, designed and supervised the study, wrote part of, and critically revised, the manuscript. All authors reviewed and approved the final manuscript.

## Conflict of Interest

The authors declare that the research was conducted in the absence of any commercial or financial relationships that could be construed as a potential conflict of interest.

## Publisher's Note

All claims expressed in this article are solely those of the authors and do not necessarily represent those of their affiliated organizations, or those of the publisher, the editors and the reviewers. Any product that may be evaluated in this article, or claim that may be made by its manufacturer, is not guaranteed or endorsed by the publisher.

## Supplementary Material

Supplementary Data Sheet 1 Methods of genetic analysis.
