# Supplementary material for: A Novel Homozygous PKP2 Variant in Severe Neonatal Non-compaction and Concomitant Ventricular Septal Defect: A Case Report
Source: Front Pediatr. 2022 Jan 4;9:801491. doi: 10.3389/fped.2021.801491 (PMC8764405; doi:10.3389/fped.2021.801491)
Supplement: Supplementary Data Sheet 1 — Methods of genetic analysis. [file Data_Sheet_1.docx]

Supplementary Material

**A Novel Homozygous *PKP2* Variant in Severe Noncompaction and Concomitant Ventricular Septal Defect: Case Report. Katanyuwong P, et al.**

# Supplementary Data

**Detailed methods of genetic analysis**

Whole exome sequencing was performed on IIumina HiSeq2500 by Macrogen® (Seoul, Republic of Korea). The raw genome sequencing data analysis including alignment to the reference sequence (GRCh37 from NCBI, February 2009), as previously described (8) Standard human phenotype ontology terms, cardiomyopathy (HP:0001638) was employed to find causative genes that matched to the patient’s phenotypes. The list of 433 genes analyzed (sarcomeric gene included) is provided in Table S1. PCR-Sanger sequencing was performed to validate the variant identified, using the following primers: forward, 5′-GAGTTGATGGCCTTGACTGA-3′, located in intron 6; and reverse, 5′- GCATCCAGTGACGTTGTAGA-3′, located in exon 7, of *PKP2* gene.

mRNA was extracted from peripheral blood, reversed transcribed to cDNA, then PCR-amplified and visualized on agarose gel, followed by DNA recovered from the gel before being subjected to Sanger sequencing, following published protocols (8). Primer sequences for the mRNA study were as follow: forward, 5′- CTGCTGCAGCTACTTTCATACA-3′ (located in exon 4) and reverse, 5′-GAGTCCGTCACATCTTCTCATCT-3′ (exon 8). Gel electrophoresis condition for mRNA study were 1% agarose gel, 0.5 TAE buffer, 100V, 35min.

Primers were designed using PRIMER3 (http://frodo.wi.mit.edu). Reference sequences used were NT_009714; NM_001005242.3, NM_001005242 for PKP2a isoform.

In brief, The PCR bands were recovered from the gel,

**Reference** (the reference number is according to that appears in the main manuscript)

8. Thongpradit S, Jinawath N, Javed A, Jensen LT, Chunsuwan I, Rojnueangnit K et al. Novel SOX10 Mutations in Waardenburg Syndrome: Functional Characterization and Genotype-Phenotype Analysis. Front Genet 2020; 11:589784.doi: 10.3389/fgene.2020.589784.

# Supplementary Figures and Tables

**TABLE S1** List of 433 cardiomyopathy-associated genes analyzed: HP0001638

**
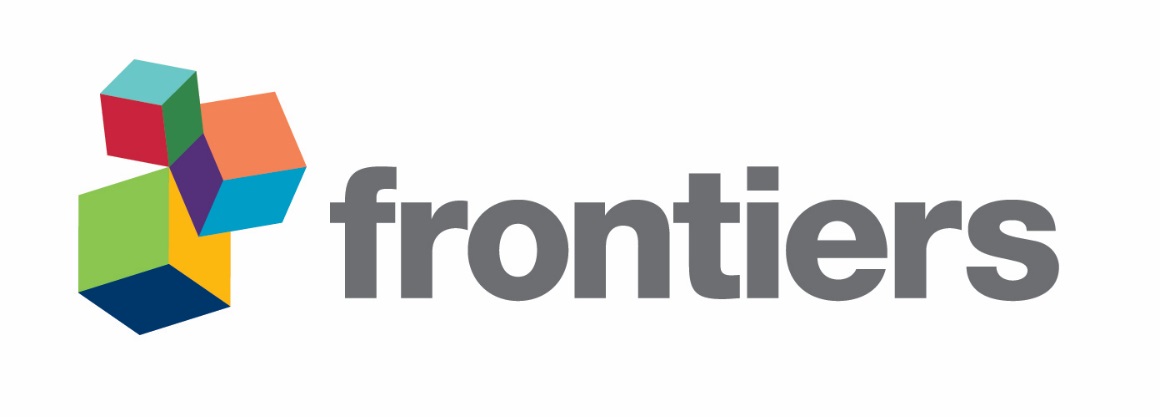
**
